# Neuroprotective effects and mechanisms of ischemic/hypoxic preconditioning on neurological diseases

**DOI:** 10.1111/cns.13642

**Published:** 2021-07-08

**Authors:** Jia Liu, Yakun Gu, Mengyuan Guo, Xunming Ji

**Affiliations:** ^1^ Laboratory of Brain Disorders Ministry of Science and Technology Collaborative Innovation Center for Brain Disorders Beijing Institute of Brain Disorders Beijing Advanced Innovation Center for Big Data‐based Precision Medicine Capital Medical University Beijing China; ^2^ Department of Neurosurgery Xuanwu Hospital Capital Medical University Beijing China

**Keywords:** hypoxia, ischemia, neurological diseases, neuroprotection, preconditioning

## Abstract

As the organ with the highest demand for oxygen, the brain has a poor tolerance to ischemia and hypoxia. Despite severe ischemia/hypoxia induces the occurrence and development of various central nervous system (CNS) diseases, sublethal insult may induce strong protection against subsequent fatal injuries by improving tolerance. Searching for potential measures to improve brain ischemic/hypoxic is of great significance for treatment of ischemia/hypoxia related CNS diseases. Ischemic/hypoxic preconditioning (I/HPC) refers to the approach to give the body a short period of mild ischemic/hypoxic stimulus which can significantly improve the body's tolerance to subsequent more severe ischemia/hypoxia event. It has been extensively studied and been considered as an effective therapeutic strategy in CNS diseases. Its protective mechanisms involved multiple processes, such as activation of hypoxia signaling pathways, anti‐inflammation, antioxidant stress, and autophagy induction, etc. As a strategy to induce endogenous neuroprotection, I/HPC has attracted extensive attention and become one of the research frontiers and hotspots in the field of neurotherapy. In this review, we discuss the basic and clinical research progress of I/HPC on CNS diseases, and summarize its mechanisms. Furthermore, we highlight the limitations and challenges of their translation from basic research to clinical application.

## Graphical abstract

IPC/HPC/RIPC exert neurprotective effects by several molecular mechanisms, including activating HIF‐1α signaling, reducing ROS, activating autophagy, anti‐apoptosis, anti‐inflammation, reducing excitotoxicity, and enhancing BBB integrity. BBB, blood brain barrier; HIF, hypoxia inducible factor; HPC, hypoxic preconditioning; IPC, ischemic preconditioning; RIPC, remote ischemic preconditioning; ROS, reactive oxygen species.

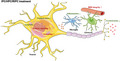

## 1 INTRODUCTION

Based on the idea that sublethal insult may induce strong protection against subsequent fatal injuries, the first hypoxic preconditioning (HPC) study took place in 1964, which confirmed HPC‐afforded tolerance of the brain against subsequent cerebral ischemic injury.^1^ In 1990, ischemic preconditioning (IPC) was reported and proved to elicit protective effects on ischemic damage.^2^ Compared with severe or pathogenic ischemic/hypoxic events, I/HPC reverses the pathological process through a milder and appropriate degree of stimulation. The brain is extremely sensitive to oxygen levels. I/HPC has been demonstrated to allow for resistance of various cerebral injuries, such as stroke, neonatal hypoxia/ischemia, and neurodegenerative diseases.^3^ Interestingly, repeated transient limb ischemia, termed “remote ischemic preconditioning (RIPC),” can also alleviate the ischemic injury of a distant organ, such as brain. RIPC has been widely studied in clinical trials in recent years and seems to be of more clinical value, because it avoids the direct ischemic/hypoxic insults to important organs.^4^ In this review, we focus on the potential therapeutic effects of I/HPC and RIPC in central nervous system (CNS) diseases, discuss the underlying protective mechanisms, and highlight the challenges of their translation from basic research to clinical application.

## 2 RESEARCH PROGRESS OF I/HPC IN CNS DISEASES

**FIGURE 1:**
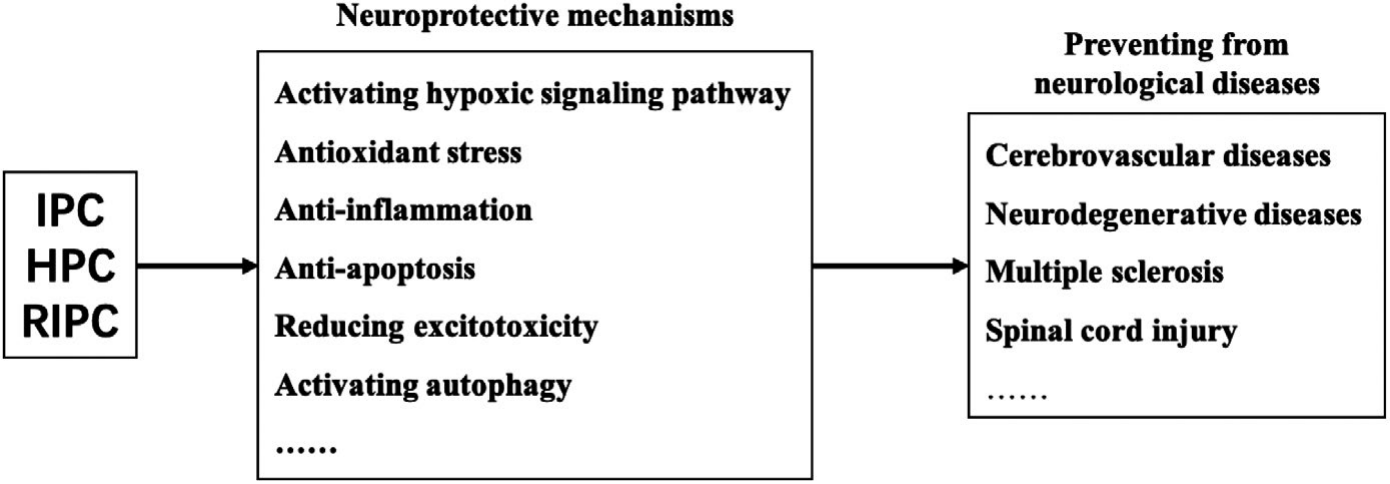
Neuroprotective mechanisms of IPC/HPC/RIPC treatment in neurological diseases. IPC/HPC/RIPC could prevent from several neurological diseases, such as cerebrovascular diseases, neurodegenerative diseases, multiple sclerosis, and spinal cord injury. There protective machenisms including activating hypoxic signaling pathway, antioxidant stress, anti‐inflammation, anti‐apoptosis, reducing excitotoxicity, and activating autophagy. HPC, hypoxic preconditioning; IPC, ischemic preconditioning; RIPC, remote ischemic preconditioning

Central nervous system diseases are comprised of cerebrovascular diseases, neurodegenerative diseases, multiple sclerosis, spinal cord injury, and others. As a potential therapeutic strategy, the protective effects of I/HPC and RIPC in CNS diseases have been extensively studied in multiple layers including in vitro cell cultures, ex vivo brain slices, in vivo experimental animal models, and clinical patients (Tables 1 and 2, Figure 1). In this section, we will introduce the research progress of I/HPC and RIPC in both clinical and preclinical CNS diseases.

**TABLE 1:** Basic research cases of neuroprotection of IPC/HPC/RIPC

Method	Subjects	Hypoxia dosage	Outcome	References
IPC	tMCAO rats	10 min of tMCAO, followed by 24 h of recovery and reperfusion	Neurological outcomes ↑ Lesion volume ↓ Apoptosis ↓	132
		30 min of tMCAO, followed by 72 h of recovery and reperfusion	Neurological outcomes ↑ Lesion volume ↓ ER stress ↓	51, 117
		5 cycles of 3 min transient occlusion of the bilateral common carotid arteries with each followed by 5 min of reperfusion	Neurological outcomes ↑ Lesion volume ↓	133
	pMCAO rats	10 min of tMCAO, followed by 24 h of recovery and reperfusion	Neurological outcomes ↑ Lesion volume ↓ Brain edema ↓ Autophagy ↑	113, 114, 134
	tMCAO mice	5 min of tMCAO, followed by 24 h of recovery and reperfusion	Lesion volume ↓	135
		12 min of tMCAO, followed by 72 h of recovery and reperfusion	Lesion volume ↓ BBB integrity ↑ Oxidative stress ↓	60, 61
		15 min of tMCAO, followed by 72 h of recovery and reperfusion	Lesion volume ↓ HIF−1α level ↑	53, 79
	pMCAO mice	7 min of tMCAO, followed by 96 h of recovery and reperfusion	Lesion volume ↓ BBB integrity ↑	124
	ICH rats	15 min of tMCAO, followed by 72 h of recovery and reperfusion	Brain edema ↓ Blood coagulation ↓	15
	forebrain ischemia gerbils	5 min forebrain ischemia, followed by 72 h of recovery and reperfusion	Neuronal apoptosis↓ Dendritic integrity ↑	136
HPC	tMCAO rats	altitude 5000 m for 3 h daily for 14 days	Lesion volume↓ Cognitive function↑ Inflammation↓	77
	tMCAO mice	8% or 11% O_2_ for 2 h or 4 h daily for 14 days	Lesion volume↓ Inflammation↓	92, 93
	tMCAO mice	8% O_2_ for 4 h, followed by 48 or 72 h of recovery	Lesion volume↓ Integrity of BBB ↑	121, 137
	Propofol‐treated rat pups	8% O_2_ for 10 min, followed by room air for a 10 min, five cycles	Apoptosis ↓	138, 139
	H‐I injury piglet	8% O_2_ for 3 h or 24 h	Brain damage ↓ HIF−1α level ↑ VEGF ↑	46
	tGCI rats	8% O_2_ for 30 min, followed by 24 h of recovery	Neurological outcomes ↑ Autophagy ↑ Apoptosis↓ Mitochondrial function↑	109, 140
	EAE mice	8% or 10% O_2_ for 14d	Integrity of BBB ↑ Inflammation↓	26, 27, 29
RIPC	tMCAO rats	Both hind limbs 4 cycles of 5 min ischemia followed by 5 min of reperfusion	Neurological outcomes ↑ Lesion volume ↓ Splenic immune response↑	9
		Left hind limb 4 cycles of 5 min ischemia followed by 5 min of reperfusion daily for 3 days	Neurological outcomes ↑ Lesion volume ↓ Apoptosis ↓	84
		Both hind limbs 3 cycles of 10 min ischemia followed by 10 min of reperfusion	Lesion volume ↓ Neurological outcomes ↑ Inflammation↓ HIF−1α and HIF−2α↓	80, 141
	tMCAO diabetic mice	Both hind limbs 3 cycles of 10 min ischemia followed by 10 min of reperfusion	Lesion volume ↓ Neurological outcomes ↑ Inflammation↓ Apoptosis↓	94, 142
	tGCI mice	left hind limb 4 cycles of 5 min ischemia followed by 5 min of reperfusion	Lesion volume ↓ Neurological outcomes ↑ Vascular dementia↓ Apoptosis↓ Oxidative stress↓	62

**TABLE 2:** Clinical study cases of neuroprotection of RIPC

Method	Subjects	Hypoxia dosage	Outcome	References
RIPC	Carotid artery stenting patients	Bilateral upper limb 5 cycles consisting of 5 min ischemia and 5 min reperfusion, twice daily for 14 days	Secondary ischemic brain injury ↓	143
	Intracranial arterial stenosis patients	Bilateral upper limb 5 cycles consisting of 5 min ischemia and 5 min reperfusion, twice daily for 300 days	Cerebral perfusion ↑ Incidence of recurrent stroke ↓ Fazekas and Scheltens scores ↓	144, 145, 146
	Subarachnoid hemorrhage patients	The upper arm 3 cycles consisting of 5 min ischemia and 5 min reperfusion for 14 days	Safe and well tolerated	13
		Lower limb 4 cycles consisting of 5 min ischemia and reperfusion for 4 times	Incidence of stroke ↓ Mortality ↓	14
	Acute ischemic stroke patients	The upper arm 5 cycles consisting of 3 min ischemia and 5 min reperfusion, twice daily for 5 days	Lesion volume ↓ Functional recovery↑	147
	Subcortical ischemic vascular dementia patients	Bilateral upper limb 5 cycles consisting of 5 min ischemia and 5 min reperfusion, twice daily for 180 days	Cognitive function↑	148
	Ischemic moyamoya disease patients	Bilateral upper limb 5 cycles consisting of 5 min ischemia and 5 min reperfusion, three times daily for 720 days	Ischemic events ↓ Cerebral perfusion ↑	149
	Small vessel disease patients	Bilateral upper limb 5 cycles consisting of 5 min ischemia and 5 min reperfusion, twice daily for 360 days	Mean flow velocity of the middle cerebral artery ↑ White matter lesion volume↓	150
	Brain tumor patients	The upper arm 3 cycles consisting of 5 min ischemia and 5 min reperfusion	Incidence of postoperative Ischemic Damage ↓ Lesion volume↓	151
	Healthy young men and women	The upper arm 4 cycles consisting of 5 min ischemia and 5 min reperfusion	Plasmic BDNF and VEGF↑ Microvascular endothelial function↑	152

### 2.1 Cerebrovascular diseases

Ischemic stroke is caused by cerebral vascular occlusion, accounting for 80% of stroke cases. Thrombolytic tissue plasminogen activator is the best strategy, but its narrow therapeutic window limits its clinical usage.^5^ Since the 1990s, several data on I/HPC have been collected in animal models of focal and global cerebral ischemia, consistently proving that regional brief ischemic/hypoxic episode exerts subsequent neuroprotection against subsequent major ischemia/hypoxia event.^6^ In rats, hypoxia exposure significantly decreased the infarct volume induced by focal permanent ischemia.^7^ Clinically, transient ischemic attack (TIA) can be regarded as a kind of IPC in situ. Patients with TIA history before an ischemic stroke were observed to have better prognosis than those without TIA history,^8^ confirming the protective effects of cerebral preconditioning. Similarly, RIPC with limb has also been found to protect against ischemic stroke in several clinical studies.^9^, ^10^, ^11^

Hemorrhagic stroke accounts for about 20% of stroke, with very limited treatment options. Rupture of intracranial aneurysms is one of the most critical reasons for subarachnoid hemorrhage (SAH), it frequently resulted in subsequent vasospasm leading to delayed cerebral ischemia (DCI) and focal neurological deficits. HPC can reduce vasospasm and DCI after SAH.^12^ RIPC was safe and well tolerated for patients with SAH,^13^ and decreased the incidence of stroke and death.^14^ IPC also protected against brain edema and blood hypo‐coagulation in intra‐cerebral hemorrhage (ICH) rats.^15^

Stem cell transplantation therapy is a hot topic in the treatment of stroke. IPC improved the curative effect of stem cell transplantation in ischemic stroke model induced by transient middle cerebral artery occlusion (tMCAO).^16^ HPC in neural stem cells and bone marrow mesenchymal stem cells (BMSCs) enhanced efficacy of stem cell therapy by promoting grafted‐cell survival in the ICH models.^17^, ^18^ Mechanistically, HPC‐treated BMSC significantly increased the expression of some key survival and regeneration factors, such as B‐cell lymphoma‐2 (Bcl‐2), brain‐derived neurotrophic factor (BDNF), and VEGF, to promote functional recovery.^18^ Taken together, these studies indicate that I/HPC is a promising strategy for therapy or combination therapy of cerebrovascular diseases, while their exact mechanism remains to be explored.

### 2.2 Neurodegenerative diseases

Neurodegenerative diseases refer to progressive dysfunction and death of selective neuronal subsets. Alzheimer's disease (AD) and Parkinson's disease (PD) are the most common neurodegenerative diseases, hypoxia also participates in their development. I/HPC is a potential approach to prevent neurodegeneration.^19^ In experimental AD animals, intermittent hypoxic training (IHT) could alleviate AD pathology and improve cognitive function by preventing neuronal loss.^20^, ^21^ This was associated with preserved cerebrovascular function through reduced oxidative stress.^20^ The idea of I/HPC was tested clinically in elderly patients with mild cognitive impairment, a precursor of AD. IHT was proved to improve cognitive function and delay the development of AD.^22^ Currently there is little evidence on the effects of I/HPC in PD, although other sorts of preconditioning such as cross‐hemispheric preconditioning seems to confer a favorable outcome in PD.^23^ Since hypoxia is closely associated with various pathogenic mechanisms of PD, we believe it is worth investigating, and is a potential direction for PD management.

### 2.3 Multiple sclerosis

Multiple sclerosis (MS) is an autoimmune disease characterized by white matter inflammatory demyelination, it is common in young and middle‐aged people.^24^ Experimental allergic encephalomyelitis (EAE) is a wildly used preclinical MS model, with similar immune pathogenesis and lesions to MS.^25^ HPC can prevent the development of EAE by decreasing leukocyte infiltration to the CNS,^26^ and microglia may play a critical role on its protective mechanisms.^27^ Furthermore, increased levels of regulatory T cells (Tregs) and anti‐inflammatory cytokine interleukin (IL)‐10 may also involve in the neuroprotective effects of HPC on EAE models.^28^ In addition to its anti‐inflammatory effects, HPC also promotes EAE recovery by promoting vascular remodeling response and enhancing blood brain barrier (BBB) integrity.^29^

### 2.4 Spinal cord injury

Spinal cord injury lead to serious dysfunction of the limbs and trunk below the injured segment. Previous studies on its treatment mainly focused on treatment timing, drug treatment, and complication treatment. In recent years, the application of physical intervention of I/HPC has attracted much.^30^ IPC reduced paraplegia incidence and neuronal damage induced by spinal cord ischemia reperfusion injury in various models by attenuating blood spinal cord barrier (BSCB) disruption,^31^ triggering spinal cord autoregulation,^32^ and upregulating endogenous antioxidant enzymes.^33^ The combination of HPC and stem cell therapy has a high translational value. HPC‐treated BMSC showed better cell survival rate and migration, along with increased neuron differentiation, enhanced paracrine effect, increased nutritional support, and improved functional recovery.^34^, ^35^ Mechanistically, HPC‐treated stem cells help shift microglial M1 to M2 polarization.^36^ A recent study also suggests that activation of hypoxia inducible factor (HIF)‐1α played a critical for the survival of BMSCs after transplantation.^37^ RIPC also attenuated motor deficits and histologic damage induced by ischemia reperfusion injury through various protective mechanisms, including suppressing BSCB disruption,^38^ upregulating antioxidant enzyme activity^39^ and preventing the increase of extracellular glutamate and subsequent excitotoxicity.^40^

### 2.5 Others

In addition to the above diseases, the protective effects of I/HPC have also been studied in various other CNS diseases. Hypobaric HPC protected animals from stress‐related depression and anxiety.^41^ HPC‐mediated molecular adaptation improved brain resistance to glutamate excitotoxicity in ethanol withdrawal.^42^ HPC can also reduce brain edema induced by alginic acid‐induced status epilepticus in rats, which may be due to stress‐related transcription factors and effector proteins.^43^ In addition, serial HPC can improve the cognitive functions in mice exposed to hypoxia.^44^

## 3 NEUROPROTECTIVE MECHANISMS OF I/HPC

**FIGURE 2:**
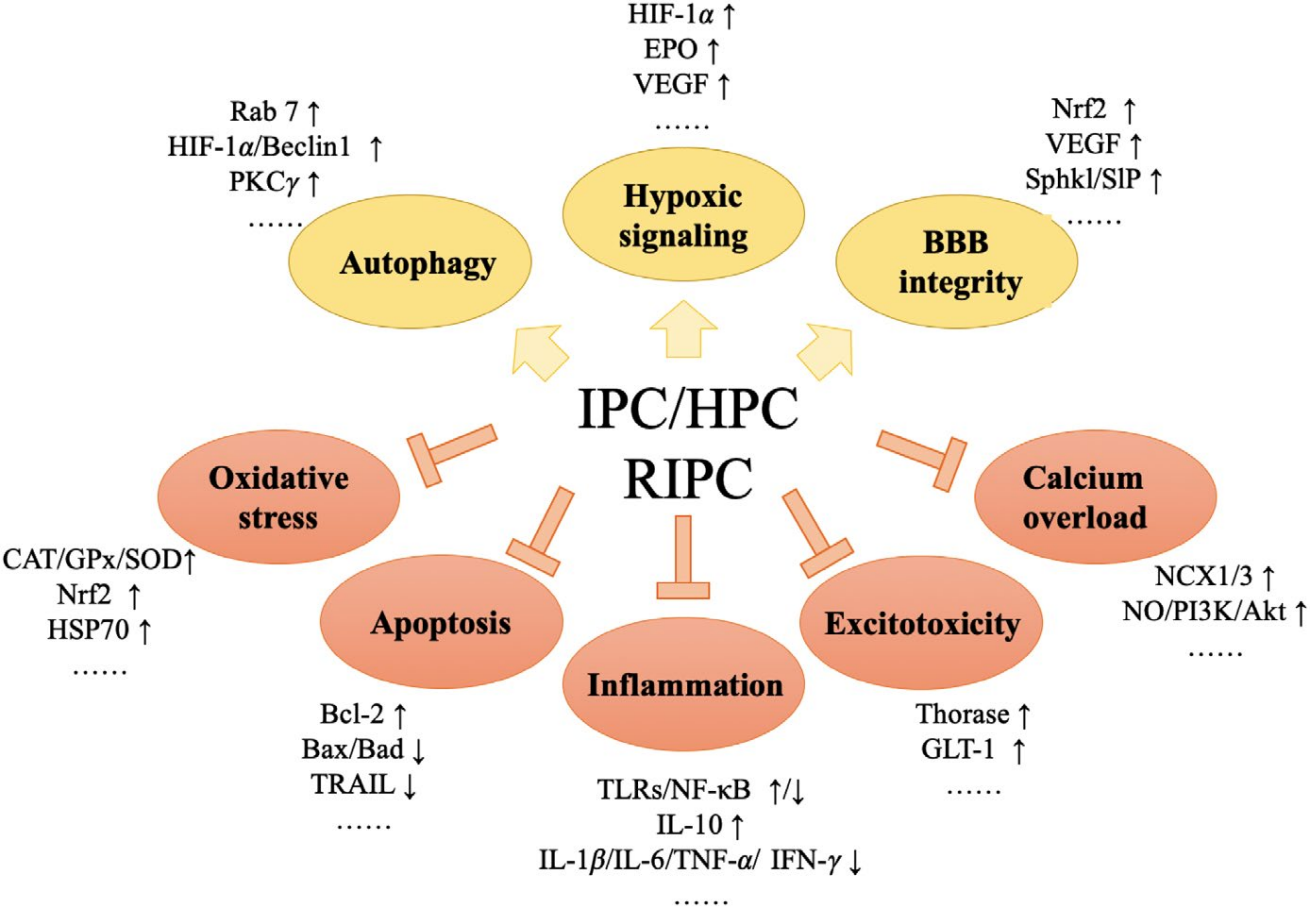
Molecular mechanisms of IPC/HPC/RIPC treatment. Various critical molecules and mechanisms are involved in neuroprotective effects of IPC/HPC/RIPC treatment. AKT, protein kinase B; BAX, Bcl‐2‐associated X; BBB, blood brain barrier; Bcl‐2, B‐cell lymphoma‐2; CAT, catalase; EPO, erythropoietin; GLT, glutamate transporter; GPx, glutathione peroxidase; HIF, hypoxia inducible factor; HPC, hypoxic preconditioning; HSP70, heat‐shock protein 70; IFN, interferon; IL, interleukin; IPC, ischemic preconditioning; NCX, Na^+^–Ca^2+^ exchanger; NF‐κB, nuclear factor‐kappa B; Nrf2, erythroid 2‐related factor 2; NO, nitric oxide; PI3K, phosphatidylinositol 3‐kinase; PKC, protein kinase C; S1P, sphingosine‐1‐phosphate; SOD, superoxide dismutase; Sphk1, sphingosine kinase; Rab, ras‐related in brain; RIPC, remote ischemic preconditioning; TLR, toll‐like receptor; TNF, tumor necrosis factor; TRAIL, TNF‐related apoptosis inducing ligand; VEGF, vascular endothelial growth factor

Conditional stimulations trigger protective responses through different sensors and signaling molecules, resulting in protective phenotypes in the brain. The mechanisms include interrelated biological pathways that minimize neuronal damage and promote the recovery through cascade of reaction. In this section, we will discuss the possible neuroprotective mechanisms related to I/HPC from the multiple aspects (Figure 2).

### 3.1 Activating hypoxic signaling pathway

**FIGURE 3:**
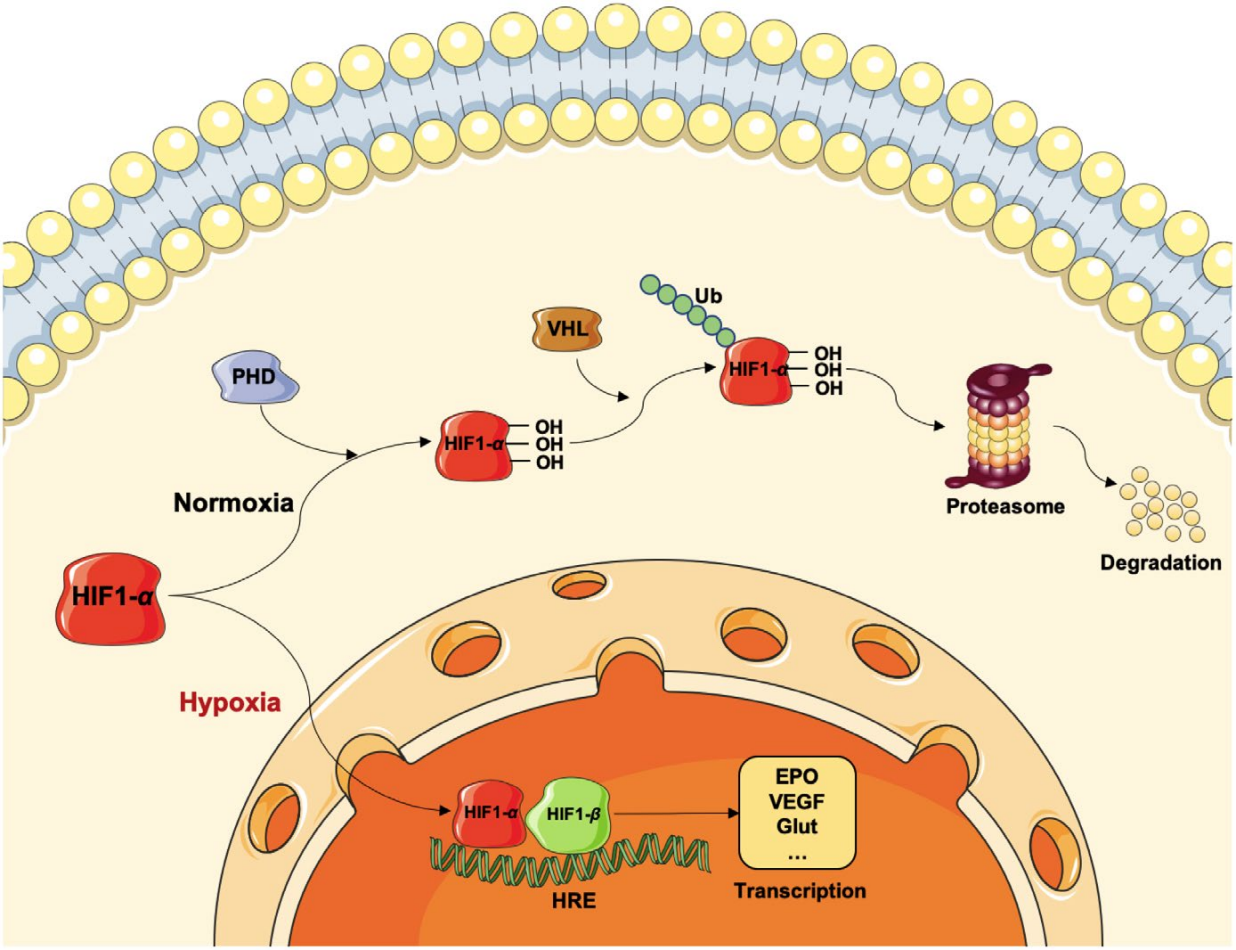
Molecular mechanisms of HIF‐1α mediated hypoxia response. Under normoxic conditions, HIF‐1α subunit is hydroxylated by PHD, which further promotes its binding with VHL complex, resulting in its ubiquitin and proteasomal degradation. Under hypoxic conditions, HIF‐1α combines with HIF‐1β to form a complex, which translocates to the nucleus and binds to HRE resulting in the transcription of multiple genes, such as EPO, VEGF, and Glut. EPO, erythropoietin; HIF, hypoxia inducible factor; HRE, hypoxia response element; PHD, proline hydroxylase; VEGF, vascular endothelial growth factor

Hypoxia inducible factor‐1 is the major molecular of hypoxic response in the brain, it composed of oxygen sensitive α subunit and structurally stable β subunit. Under physiological conditions, HIF‐1α subunit is hydroxylated by proline hydroxylase (PHDs), which further promotes its binding with Von Hippel‐Lindau (VHL) complex, resulting in its ubiquitination and proteasomal degradation. Under hypoxic conditions, HIF‐1α combines with HIF‐1β to form a complex, which translocates to the nucleus and binds to the hypoxia response element on the target gene, resulting in the transcriptional activation of multiple genes, such as erythropoietin (EPO) and VEGF (Figure 3).^45^

HPC could significantly increase HIF‐1α level and its nuclear translocation, and whereby increase the expression of its target gene VEGF in neurons, endothelial cells, and astrocytes.^46^ Some other HIF‐1 target genes were also required for HPC‐induced tolerance, such as cyclin‐dependent kinase inhibitor p21, whose deficiency abolished the neuroprotection of HPC.^47^ Maintaining intracellular Ca^2+^ homeostasis is crucial to prevents Ca^2+^‐associated cell damage, IPC increases the expression of Na^+^‐Ca^2+^ exchanger (NCX) 1, which helps in this regard through HIF‐1 signaling.^48^ Another mechanism via which IPC modulates Ca^2+^ homeostasis is through NCX1 and NCX3 upregulation mediated by nitric oxide (NO)/phosphatidylinositol 3‐kinase (PI3K)/protein kinase B (Akt) signaling.^49^, ^50^ Sumoylation of NCX3 stabilizes NCX3, and is regarded as a potential target in IPC‐induced neuroprotection.^51^ Implantation of HPC‐treated hematopoietic stem cells improved stroke outcomes through promoting neuroplasticity mediated by HIF‐1α induction.^52^ Interestingly, unlike hypoxia‐dependent mechanism in neurons, astrocytes enhance HIF‐1α expression through P2X7‐receptor‐dependent mechanism.^53^

### 3.2 Antioxidant stress

Under normal circumstances, the body has an effective endogenous antioxidant defense system. Oxidative stress is a state of imbalance between oxidation and antioxidation in the body, it can be induced by excessive production of reactive oxygen species (ROS) or decreased ability of scavenging ROS. I/HPC and RIPC could decreases the levels of ROS and increases the levels of antioxidant enzymes to prevent from neuronal injury. IPC could increase catalase (CAT), glutathione peroxidase (GPx) and thioredoxin 2 activities to eliminate the excessive ROS in the hippocampal cornu ammonis (CA) 1 region.^54^ Similarly, HPC increases activities of superoxide dismutase (SOD) and GPx in ischemic brain injury model.^55^ RIPC reduces cerebral oxidative damage by increasing activity of CAT and reducing methane dicarboxylic aldehyde levels.^56^, ^57^ RIPC improve memory and cognitive function by enhancing SOD activity after hippocampal ischemia.^58^

Despite regulating the above antioxidant enzymes, I/HPC and RIPC also reduced cerebral injury through antioxidant stress via various critical signaling pathways. Transcription factor erythroid 2‐related factor 2 (Nrf2) is a master redox regulator. HPC protects the brain against traumatic damage by upregulating Nrf2 level and suppressing oxidative stress damage.^59^ In MCAO model, IPC alleviated motor deficits and cognitive impairment, accompanied by Nrf2 pathway activation, while these protective effects of IPC were abolished in Nrf2 knockout mice.^60^ Nrf2 also played a critical role in IPC‐mediated blood‐brain barrier (BBB) preservation and neuroprotection.^61^ Similarly, RIPC prevented mice from vascular dementia by increasing Nrf2 level to decrease oxidative stress.^62^ Heat‐shock protein 70 (HSP70) is a cellular defense factor under stress, which can be upregulated by IHPC stimulus.^63^, ^64^ RIPC could mediate brain ischemic tolerance through activation of p38 mitogen‐activated protein kinase by upregulating HSP70 expression ^65^ and HIF‐1α/AMPK/HSP70 pathway.^66^

In addition to the above critical molecules, I/HPC also played an antioxidant role through other ways. Mitochondrial respiratory chain is the main source of cellular ROS. In astrocytes, IPC promotes localization of Nrf2 on the mitochondrial outer membrane, thus preventing abnormal supercomplex formation and maintaining mitochondrial function.^67^ IPC also regulate mitochondrial NAD+/NADH ratio through regulating nicotinamide phosphoribosyltransferase activity via protein kinase C (PKC) ɛ activation.^68^ IPC facilitates the repair of oxidative DNA damage induced by ischemic injury through inducible DNA base‐excision repair.^69^ RIPC protected neurons and mitochondria from oxidative damage in the porcine model of hypothermic ischemic insult.^70^ It also reduced systemic oxidative stress by about 80% represented by lymphocytic DNA damage, and reduced circulating glutamate levels in rodents.^71^ Furthermore, plasma from RIPC donor rabbits could also protect neural stem cells from oxidative stress and apoptosis through induction of thioredoxin,^72^ and the involvement of adenosine A1 receptors also play a role.^73^

### 3.3 Anti‐inflammation

**FIGURE 4:**
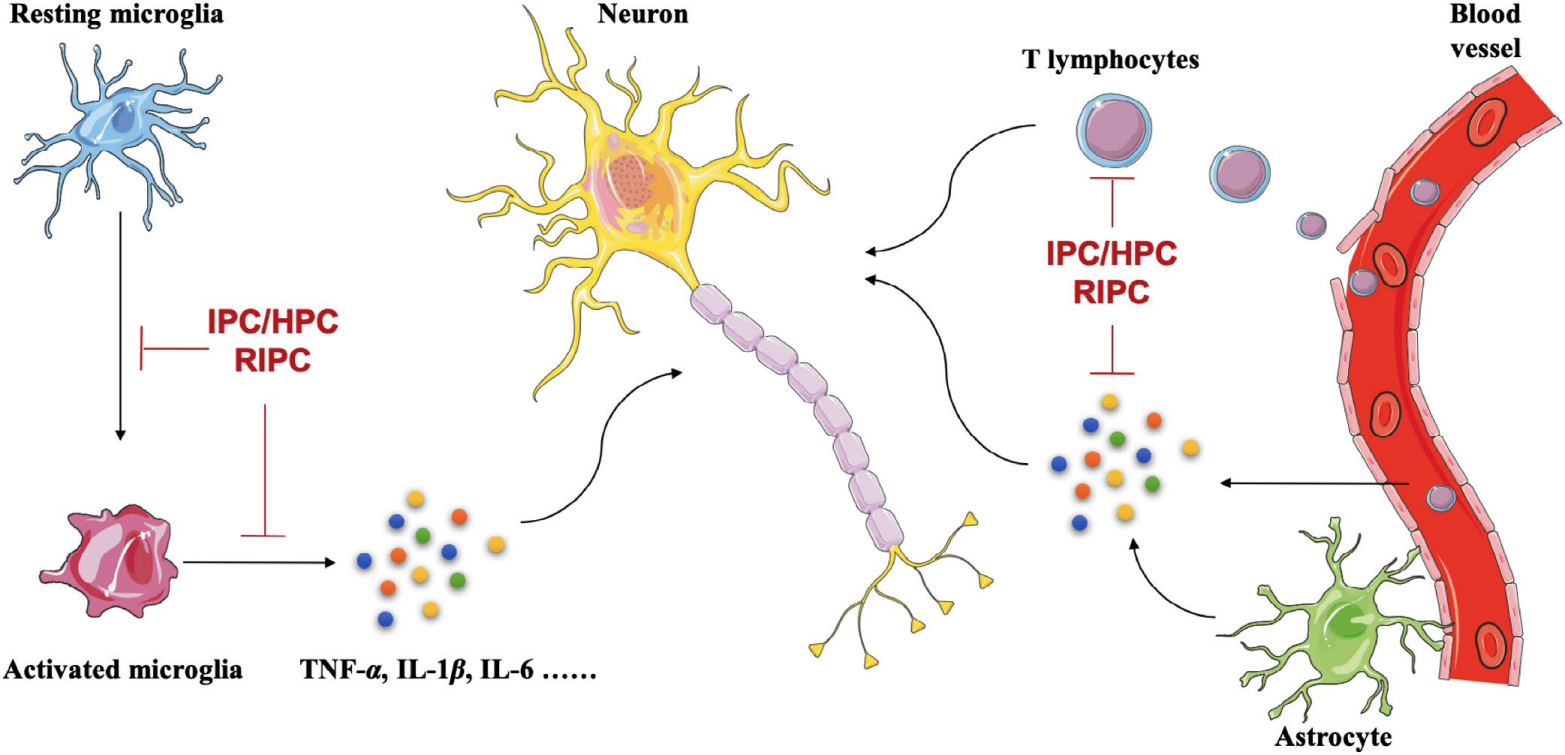
IPC/HPC/RIPC relieve neuroinflammation induced through central and peripheral immune cells. Neuroinflammation is involved in the pathogenesis of many neurological diseases. In the CNS, microglia or astrocytes activation could result in the release of inflammatory factors, such as TNF‐α, IL‐1β, and IL‐6. In addition, peripheral immune cells such as T lymphocytes and monocytes also infiltrate into CNS through BBB, which is usually destructive in most neurological diseases. The above process could be relieved by IPC/HPC/RIPC. BBB, blood brain barrier; CNS, central nervous system; HPC, hypoxic preconditioning; IL, interleukin; IPC, ischemic preconditioning; RIPC, remote ischemic preconditioning; TNF, tumor necrosis factor

Neuroinflammation is a double‐edged sword, appropriate duration and extent facilitate clearance of dead tissue and restoration of homeostasis, but excessive inflammatory response aggravates brain damage and affect long‐term neurological outcome. I/HPC and RIPC modulated immune response at various layers, including molecular, cellular, and systemic mechanisms to prevent from secondary neural injury (Figure 4).^74^

As the brain resident immune cells, microglia are among the most important cells which orchestrate neuroinflammatory response.^75^, ^76^ HPC could suppress microglia abnormal activation and subsequent inflammatory responses after hypoxia‐ischemia insults.^77^ In addition, conditioned medium from HPC‐treated BMSCs could switch microglia toward anti‐inflammatory polarization and alleviate microglia‐induced injury by inhibiting the levels of pro‐inflammatory cytokines, such tumor necrosis factor (TNF)‐α, and upregulating anti‐inflammatory cytokines, such as IL‐10.^78^ Interestingly, IPC could induce cortical microglial proliferation dependent on fractalkine signaling.^79^ RIPC inhibited inflammation by decreasing the levels of IL‐1β, IL‐6, and interferon‐γ in the ischemic brain.^80^ Astrocytes are another type of glial cells which also exert immune regulation,^81^ it mediate inflammatory effects by releasing neurotransmitters such as glutamate, and cytokines such as TNF‐α. IPC could reduce the damage of ischemia reperfusion effectively by reducing the release of astrocytic glutamate, which was further enhanced with astrocytic gap junction blockade.^82^

Aside from cellular mediators, several signaling pathways also participate in the anti‐inflammatory effects of preconditioning treatment. Nuclear factor‐kappa B (NF‐κB) is a key player in mediating inflammation, exhibits a significant role in cerebral ischemic tolerance induced by I/HPC and RIPC. IPC could activate PKCε and ERK1/2 to promoted NF‐κB translocation to nucleus,^83^ and RIPC‐mediated ischemic tolerance by activating NF‐κB pathway through interaction with Notch1 pathway.^84^ On the contrary, there were also lots of studies suggested IPC suppressed NF‐κB activation.^85^, ^86^ IPC downregulated NF‐κB expression through inhibiting PI3K/Akt and ERK1/2 signaling pathways. As master regulators of innate immunity, toll‐like receptors (TLRs) play a critical role in CNS inflammatory response.^87^ IPC reduces cerebral ischemic injury by inhibiting of the TLR4/NF‐κB signaling pathway.^88^ Astrocytic TLR3 reprogramming also participates in IPC‐induced anti‐inflammation and ischemic tolerance.^89^

I/HPC and RIPC also creates anti‐inflammatory effects through affecting chemotaxis of the peripheral immune cells.^90^ HPC‐induced ischemic tolerance by decreasing early leukocyte infiltration dependent on a delay in C‐C motif chemokine ligand (CCL) 2 expression.^91^ In addition, HPC‐induced chemokine (C‐X‐C motif) ligand 12 upregulation which suppressed leukocyte infiltration in tMCAO mice.^92^ HPC also activated a novel immunosuppressed B‐cell phenotype to exert anti‐inflammatory effects in tMCAO mice model.^93^ RIPC increased B cell in peripheral blood^90^ and reduced T‐cell infiltration into the ischemic brain, accompanied by increased p‐ERK expression.^94^ Another study also suggested RIPC exerted neuroprotection against cerebral ischemia mainly by modulating the spleen‐derived lymphocytes.^9^

### 3.4 Anti‐apoptosis

Apoptosis is an active and orderly cell death process,^95^ I/HPC and RIPC could protect against brain injury by inhibiting apoptosis. The expression of anti‐apoptotic genes increased after HPC treatment in hippocampal slice cultures.^96^ IPC could downregulate pro‐apoptosis protein BCL‐2‐associated X (Bax) level, but upregulate anti‐apoptosis protein Bcl‐2 level, and further decrease cleaved caspase‐9 and caspase‐3.^85^, ^97^ It also blocked the ischemia‐induced mitochondrial translocation of Bad, a Bcl‐2 family member, via PI3K/Akt signaling, inhibiting apoptosis of CA1 pyramidal cells.^98^ IPC prevented the opening of mitochondrial permeability transition pore (mPTP) and the releasing of cytochrome c mediated by nitrite.^99^ Similarly, RIPC also decreased apoptosis of hippocampal neurons by improving the integrity of the mitochondrial membrane and inhibiting mPTP opening.^100^ Endoplasmic reticulum (ER) stress is a strong inducer of apoptosis. IPC inhibited ER stress‐induced apoptosis through protein kinase RNA (PKR) like ER kinase pathway.^101^ IPC could also downregulate TNF‐related apoptosis inducing ligand, a critical death receptor.^102^

### 3.5 Reducing excitotoxicity

Excitotoxicity is a toxic process mainly caused by excessive excitatory neurotransmitter glutamate.^103^ Thorase is important to maintain mitochondrial function and regulate surface glutamate receptor activity. IPC‐induced thorase expression to provide neuroprotection against N‐methyl‐D‐aspartic acid (NMDA) receptor‐mediated excitotoxicity.^104^ IPC‐treated astrocytes could also confer ischemic tolerance to neurons associated with increased neuronal tolerance to NMDA.^105^ Glutamate homeostasis in the CNS is maintained through uptake of excessive glutamate by excitatory amino acid transporter known as glial glutamate transporter (GLT)‐1. GLT‐1 is mainly located on astrocytes, and has been regarded as a potential therapeutic target in the treatment of brain ischemic injury, IPC can reduce glutamate excitotoxicity by upregulation of GLT‐1 activity in glial cells, thus inducing cerebral ischemic tolerance.^106^ HPC also reversed the downregulation of GLT‐1 protein caused by global cerebral ischemia.^107^

### 3.6 Activating autophagy

Autophagy is an evolutionarily conservative process crucial for cell survival, in the circumstances of hunger, infection and stress, autophagy contributes to homeostasis by removing aggregated proteins and damaged organelles, rapidly providing fuel supply for energy, and delaying cell death. In ischemia model, HPC increased the production and degradation of autophagosomes and resisted to subsequent fatal injury.^108^ HPC also promoted autophagosome maturation by activating ras‐related in brain 7, a lysosome‐related protein, to protect against global cerebral ischemia‐induced injury.^109^ HIF‐1α/Beclin1 signaling pathway activation was also involved in autophagy induction during HPC treatment.^110^ Conventional PKCγ signaling molecules especially PKCγ‐synapsin pathway were proved to facilitate HPC‐mediated protection,^111^ and PKCγ could modulate neuron‐specific autophagy through the Akt‐mTOR pathway MCAO mice model.^112^ Similarly, IPC increased the levels of autophagy related proteins, including microtubule‐associated protein 1 light chain (LC) 3 II and beclin1, which were suppressed by autophagy inhibitors 3‐methyladenine and bafilomycin A1, and enhanced by autophagy agonist rapamycin, confirming the critical role of autophagy in IPC.^113^ IPC also protected against neuronal injury via ER stress‐induced autophagy, proved by abolished neuroprotection with ER stress inhibitor salubrinal.^114^

### 3.7 Others

Besides the above protective mechanisms, other mechanisms associated with I/HPC include but not limited to improving synaptic plasticity, modulating Ca^2+^ homeostasis, and preserving BBB function. IPC improved synaptic plasticity by increasing BDNF mRNA expression dependent on PKCɛ activation.^115^ Meanwhile, IPC‐induced PKCɛ activation enhances GABA release, contribute to monoamine balance of the brain.^116^ Ca^2+^ homeostasis was maintained by IPC through modulating the interaction of the ER‐located Ca^2+^ sensor stromal interacting molecule 1 with the plasma membrane channel ORAI1,^117^ as well as activation of the NO/PI3K/Akt pathway.^49^ Impairment of BBB integrity is a critical event in the pathogenesis of ischemic/hypoxic injury.^118^ HPC could induce de novo formation of cerebral collaterals, which lessens the severity of a subsequent stroke event.^119^ HPC also upregulates the expression of vascular genes, whereby increasing vascular density and cerebral blood flow.^120^ HPC could also active sphingosine kinase 2 in a region‐specific manner, whose products sphingosine‐1‐phosphate is critical for vascular functioning.^121^, ^122^, ^123^ Astrocytes are proved to be major mediators in IPC‐mediated BBB preservation in vitro.^124^ HPC could also help restore the maturation capacity in oligodendrocyte precursor cells in neonatal rats subjected to hypoxia/ischemia insults.^125^

In addition to the proteins involved in the above mechanisms, some other molecules have also been proposed as potential targets of I/HPC. Heme oxygenase 1 (HO1), an anti‐oxidant enzyme, is significantly increased by IPC, and HO1 knockout could abolish IPC‐induced protective effects on ischemic brain injury, indicating its critical role in IPC.^126^ Considerably, IPC protects hippocampal pyramidal neurons from ischemic injury by HO1‐mediated suppression of oxidative damage.^127^ An apoptotic inhibitory molecule, cellular inhibitor of apoptosis 1, was also implicated in IPC in neurons and endothelial cells.^128^ Epigenetic studies by microarray analysis suggested that methyl‐CpG binding protein 2 was also a prominent target in IPC‐induced tolerance.^129^

## 4 LIMITATIONS AND CHALLENGES

Although both of I/HPC and RIPC have shown considerable protective effects in lots of CNS diseases, there are still many limitations and challenges to translate their clinical applications from basic research. Firstly, heterogeneity of population subgroups needs to be considered. Different individual factors, such as age, gender, race, and comorbid medical conditions require different degrees of conditioning treatment to induce optimal stimulate. In addition to these individual factors, different diseases should also be treated in different ways. In other words, the optimal duration and frequency of pretreatment stimuli vary from disease to disease, and different hypoxic levels, duration, and onset cycle may have very different effects. Secondly, timeliness is also a main limitation of I/HPC and RIPC, because their benefits usually last only a few days, while the onset of diseases is unpredictable. Therefore, patients should be stratified based on their risk for each individual disease, and different therapeutic strategy should depend on the pathogenesis individually. The combination therapy with preconditioning and postconditioning may be a promising direction.^130^ In addition, specific biomarkers that respond to preconditioning treatment may be useful to guide optimal therapeutic strategy and further assess efficacy. Furthermore, cross adaptation or cross tolerance is also a growing field of interest, it refers to the phenomenon that one type of conditioning could establish tolerance toward another type of injury.^131^ It suggests that I/HPC may play a role in more fields, which need to be further explored. Finally, although application of RIPC is practical in high‐risk population, while it maybe not practical to induce ischemia/hypoxia in healthy humans, so more mechanistic studies can help us achieve the protective effect through other methods, such as pharmacological treatment.

## 5 SUMMARY

Due to the complicated etiology of neurological diseases, the current treatment strategies are still limited. In addition, many neurological diseases come on gradually, so once diagnosed, the best treatment window is missed. Therefore, preconditioning therapy is a promising direction in the treatment of neurological diseases. Specific molecular mechanisms of I/HPC or RIPC need to be further explored, and their limitations and challenges need to be addressed as well.

## CONFLICT OF INTEREST

The authors declare no conflict of interest.

## Data Availability

Data sharing is not applicable to this article as no new data were created or analyzed in this study.
